# Exercise Training as a Treatment for Cardiometabolic Risk in Sedentary Adults: Are Physical Activity Guidelines the Best Way to Improve Cardiometabolic Health? The FIT-AGEING Randomized Controlled Trial

**DOI:** 10.3390/jcm8122097

**Published:** 2019-12-01

**Authors:** Francisco J. Amaro-Gahete, Alejandro De-la-O, Lucas Jurado-Fasoli, Borja Martinez-Tellez, Jonatan R. Ruiz, Manuel J. Castillo

**Affiliations:** 1 EFFECTS-262 Research Group, Department of Medical Physiology, School of Medicine, University of Granada, 18016 Granada, Spain; delao@ugr.es (A.D.-l.-O.); juradofasoli@ugr.es (L.J.-F.); mcgarzon@ugr.es (M.J.C.); 2 PROmoting FITness and Health through Physical Activity Research Group (PROFITH), Sport and Health University Research Institute (CIDS), Department of Physical Education and Sports, Faculty of Sport Sciences, University of Granada, 18011 Granada, Spain; borjammt@ugr.es (B.M.-T.); ruizj@ugr.es (J.R.R.); 3 Department of Medicine, Division of Endocrinology, and Einthoven Laboratory for Experimental Vascular Medicine, Leiden University Medical Center, 2333 Leiden, The Netherlands

**Keywords:** cholesterol, concurrent training, HIIT, insulin resistance, insulin sensitivity, triglycerides, whole-body electromyostimulation

## Abstract

This 12-week randomized controlled trial investigates the effects of different training modalities on cardiometabolic risk in sedentary, middle-aged adults, and examines whether alterations in cardiometabolic risk are associated with changes in those health-related variables that are modifiable by exercise training. The study subjects were 71 middle-aged adults (~54 years old; ~50% women) who were randomly assigned to one of the following treatment groups: (1) no exercise (control group), (2) concurrent training based on international physical activity recommendations (PAR group), (3) high intensity interval training (HIIT) group, or (4) HIIT plus whole-body electromyostimulation (HIIT+EMS group). A cardiometabolic risk score was calculated based on the International Diabetes Federation’s clinical criteria. A significant reduction in cardiometabolic risk was observed for all exercise training groups compared to the control group (all *p* < 0.05), which persisted after adjusting potential confounders (all *p* < 0.05). However, the HIIT+EMS group experienced the most significant reduction (*p <* 0.001). A significant inverse relationship was detected between the change in lean mass and the change in cardiometabolic risk (*p =* 0.045). A 12-week exercise training programs-especially the HIIT+EMS program-significantly reduced cardiometabolic risk in sedentary, middle-aged adults independent of sex, age, and cardiorespiratory fitness.

## 1. Introduction

In recent decades, the worldwide prevalence of cardiovascular and chronic non-communicable metabolic disease has dramatically increased among young, middle-aged and elderly adults [1,2]. Metabolic syndrome, obesity and type II diabetes mellitus all strongly increase the risk of cardiovascular disease [3]. Changes in body composition (i.e., greater fat mass, larger amounts of visceral adipose tissue, and less lean mass) [4], hypertension [5], impaired glucose metabolism (i.e., the development of insulin resistance) [6], altered lipid metabolism (i.e., raised plasma triglycerides, total cholesterol and low-density lipoprotein cholesterol [LDL-C], and reduced high-density lipoprotein cholesterol [HDL-C]) [7], low cardiorespiratory fitness [8], and an unhealthy lifestyle [9] all increase this risk.

The potential benefits of physical exercise on cardiometabolic health (independent of age, sex, and other biological factors) have been well-documented [10]. International physical activity guidelines for health promotion establish that the adult population should complete at least 150 min per week of moderate intensity aerobic exercise, or 75 min per week of vigorous intensity aerobic exercise, combined with resistance training twice per week [11,12]. Previous studies have shown that concurrent training (i.e., the combination of endurance and resistance training) can substantially improve the cardiometabolic profile of healthy individuals, as well as those of patients with metabolic abnormalities [13,14]. However, the majority of people in developed societies do not meet current physical activity recommendations, a lack of time being the most commonly cited obstacle [15]. Novel, time-efficient training methods have, however, recently emerged.

Low-volume, high intensity interval training (HIIT) requires relatively little time and seems capable of inducing improvements in cardiometabolic risk similar to—or even better than—that achieved by traditional endurance training at moderate intensity (which requires 400% more commitment in terms of time) [16,17]. However, there have been no studies comparing the effects of concurrent training and HIIT on the cardiometabolic risk profile of healthy or unhealthy individuals.

Whole-body electromyostimulation (WB-EMS), which simultaneously stimulates up to 16 muscle groups (with different intensities per group) has recently arisen as an exercise training modality with the promise of being able to significantly improve the cardiometabolic health of elderly men [18]. We recently reported that an HIIT program plus WB-EMS enhanced the physical fitness and body composition of sedentary, middle-aged adults [19,20]. However, it remains unknown whether an HIIT program combined with WB-EMS can improve the cardiometabolic profile in previously sedentary, middle-aged adults, and whether any hypothetical improvements would be greater than those obtained by HIIT alone, or those obtained by concurrent training based on international physical activity guidelines for health promotion [11,12].

The present work investigates the effects of these training modalities on cardiometabolic risk in previously sedentary, middle-aged adults, and examines whether alterations in cardiometabolic risk are associated with changes in health-related outcomes that are modifiable by exercise training (i.e., body composition, physical fitness, etc.).

## 2. Experimental Section

### 2.1. Ethics Statement and Reporting Philosophy

This study was performed as part of the FIT-AGEING project, a full description of which is available at clinicaltrial.gov: ID: NCT03334357 (07/11/2017) [21]. The present study protocol was approved by the Ethics Committee on Human Research of the Regional Government of Andalucía [0838-N-2017], and complies with the latest revision of the Declaration of Helsinki. Written, informed consent was obtained from all potential participants prior to their inclusion in the project. The present text adheres to the CONSORT statement for improving the reporting of parallel group randomized trials (EQUATOR Network; Table S1) [22].

### 2.2. Study Subjects and Treatment Groups

A total of 89 individuals (~50% women) aged 40–65 years were recruited (via local media, social networks and posters) to this 12-week, randomized, parallel group, controlled trial. The inclusion criteria were: (i) being sedentary (exercising <20 min on <3 days/week), (ii) having a stable body weight over the previous 12 weeks (body weight changes <5 kg), and (iii) having no chronic metabolic disease (e.g., diabetes mellitus type II), cardiovascular disease, cancer, or any problem that might be aggravated by exercise training.

### 2.3. Exercise Training

The study was organized in two waves (September–December 2016 and September-December 2017) due to reasons of feasibility and practicality, and to avoid any potential seasonal bias. Using simple randomization software [23] the subjects were assigned to one of the following treatment groups: (1) no exercise (control group), (2) concurrent training based on international physical activity recommendations (PAR group), (3) high intensity interval training (HIIT group), or (4) high intensity interval training plus whole-body electromyostimulation (HIIT+EMS group). The team interpreting the results was blinded to the randomization process. To improve the replicability and transparency of the methodology followed, these exercise programs follow the norms of the Consensus on Exercise Reporting Template (CERT; Table S2).

Individuals in the non-exercise control group were asked to maintain their physical activity levels and dietary habits over the 12-week study period. In addition, they were provided with general recommendations about a healthy lifestyle.

The PAR group subjects participated in three training sessions per week for all 12 weeks. In total, this involved 150 min/week of aerobic training at 60–65% of their heart rate reserve organized in 10 min bouts, and using a treadmill, a cycloergometer, and/or an elliptical ergometer. They also completed 60 min/week resistance training (global strength exercises including bench presses, lateral pull downs, dead lifts, and squats, among others) at 40–50% of their one-maximum repetition. A recovery period of 48 h was allowed between training sessions.

The HIIT group subjects participated in two training session each week, following a long interval HIIT (HIIT-LI) and a short interval HIIT (HIIT-SI) protocol [24,25]. For the HIIT-LI component, they exercised for 40–65 min/week at >95% of their maximum oxygen uptake (VO_2_max), walking on a treadmill with a personalized slope. For the HIIT-SI component they undertook weight-bearing circuit training at level 6–9 on a perceived maximum effort scale [ranged from 0 to 10] [26]. A recovery period of 72 h was allowed to elapse between training sessions.

The HIIT+EMS subjects performed exactly the same exercise training as the HIIT group in terms of frequency, volume, intensity, type of exercise and periodization, but with additional WB-EMS during exercise. Given that the subjects had never trained with WB-EMS, a preliminary adaptational period was allowed to prevent any side effects [27]. Pulses were rectangular, bipolar and symmetrical at a frequency of 15–20 Hz in HIIT-LI and 35–75 Hz in HIIT-SI, and at an intensity of 100 mA in HIIT-LI and 80 mA in HIIT-SI. The impulse width was 200–400 µs. The duty cycle was 99% for HIIT-LI and 50–63% for HIIT-SI. All WB-EMS tools was provided using a Wiemspro^®^ device (Wiemspro, Malaga, Spain), following the manufacturer’s instructions.

All sessions started with a dynamic, standardized warm-up (10 min), and finished with a cooling-down protocol (active global stretching). Detailed information regarding the dose and intensity of each training intervention is available elsewhere [19,20,28]. All sessions were performed in small groups (2–6 subjects), strictly monitoring subject safety and their adherence to the required training intensity and volume. All sessions were conducted at the Centro de Investigación Deporte y Salud (CIDS), University of Granada (Spain), and were monitored by exercise professionals with a degree in Sports Sciences. Training session attendance was recorded daily; repeat sessions were made available on alternative days to facilitate the recovery of any missed. A 90% minimum attendance rate was fixed for data use.

### 2.4. Outcomes

Anthropometry and body composition. Weight and height were measured using a SECA model 799 electronic scale and stadiometer (SECA, Hamburg, Germany). Body mass index (BMI) was determined as weight (kg)/height (m)^2^. Waist circumference was assessed at the midpoint between the iliac crest and the last rib. Body composition was assessed using a Discovery Wi dual-energy X-ray absorptiometer (Hologic, Inc., Bedford, MA, USA), obtaining fat mass and lean mass following the manufacturer’s recommendations. 

Blood pressure. Blood pressure was determined in the right arm after a 30 min rest in a supine position, using an Omrom^®^ HEM 705 CP automatic monitor (OMROM Health-Care Co., Kyoto, Japan), following the recommendations of the European Heart Society [29] A minimum of three measurements were taken 1 min apart, and the mean value calculated.

Blood samples. Venous blood samples were taken in fasting conditions [i.e., ~12 h] from the antecubital vein and collected in ethylenediamine tetra-acetic acid-containing tubes using the Vacutainer SST system (Becton Dickinson, Plymouth, UK) All samples were centrifuged at 4000 rpm for 7 min at 4 °C, and aliquots of plasma stored at −80 °C until analysis. Plasma glucose, total cholesterol, HDL-C, triglycerides, alanine transaminase (ALT), and γ-glutamyl transferase (γ-GT) were determined using an AU5800 absorption spectrophotometer (Beckman Coulter, Brea, CA, USA). Plasma insulin was assessed by chemiluminescence immunoassay using a UniCel DxI 800 device (Beckman Coulter, Brea, CA, USA). LDL-C was determined using the equation (total cholesterol) − (HDL-C) − 0.45 * (triglycerides). Blood samples were collected after 72–96 h of the last bout of exercise in the post-intervention assessment. 

Cardiometabolic risk score. The International Diabetes Federation [30] has proposed clinical criteria-waist circumference, blood pressure, and plasma glucose, HDL-C, and triglyceride concentrations-defining cardiometabolic risk. Sex-specific cardiometabolic risk scores were calculated based on these criteria. Each variable was standardized as follows: standardized value = (value − mean)/standard deviation. The HDL-C standardized values were multiplied by −1 to represent increasing values as directly proportional to the risk score. The final score was determined as the sum of the five standardized scores divided by 5. The cardiometabolic risk score is a continuous variable with a mean of 0 and a standard deviation of 1 by definition, with lower scores denoting a more favorable profile.

Fatty liver index. The fatty liver index is a validated surrogate marker of non-alcoholic fatty liver disease [31]. This was calculated from the body mass index, waist circumference, triglycerides, and γ-GT using the following equation:

$$Fatty\;liver\;index=\left(e^{0.953*loge\left(triglycerides\right)+0.139*body\;mass\;index+0.718*loge\left(\gamma -\mathrm{GT}\right)+0.053*\mathrm{waist}\;\mathrm{circunference}-15.745)}\right)*100$$

Quantitative insulin sensitivity check index (QUICKI). This was calculated as the inverse of the sum of the logarithms of the plasma insulin (UI/mL) and plasma glucose (mg/dL) [32] concentrations.

Homeostatic model assessment of insulin resistance index (HOMA). This was determined as plasma insulin (UI/mL) × plasma glucose (nmol/L)/22.5 [33].

Dietary intake. Dietary intake was recorded via three non-consecutive 24 h recall records, collected by a qualified nutritionist. Total energy intake and the macronutrient distribution were calculated using EvalFINUT^®^ software, which makes use of the U.S. Department of Agriculture and the Spanish BEDCA (Base de Datos Española de Composición de Alimentos) databases.

Cardiorespiratory fitness. VO_2_max was determined by indirect calorimetry using a maximum graded treadmill test following the modified Balke protocol [34] (explained in detail elsewhere) [19]. VO_2_max was deemed reached when: (i) the respiratory exchange ratio was >1.1, (ii) a plateau in VO_2_ (change of <100 mL/min in the last 30 s of the test) had been reached, and (iii) a heart rate of within 10 bpm of the age-predicted maximum was observed [35]. When these criteria were not met, peak oxygen uptake during the test was recorded [35].

### 2.5. Statistical Analysis

Explanations of the statistical power requirements for the present work are available elsewhere [19,20,21,28]. Briefly, it was assumed that 25% of subjects would drop-out over the 12-week study period. Based on a pilot study, statistical power was fixed at 85% for detecting post-intervention cardiometabolic risk improvements of 10–15% (type 1 error = 0.05) [4]. A total of 14 subjects per group were necessary to meet these criteria.

Data are expressed as means (standard deviation), unless otherwise stated. Data normality was confirmed using the Shapiro-Wilk test, visual histograms and Q-Q plots. Between-group baseline differences were examined by one-way analysis of variance (ANOVA). Given that the aim of the study was to examine the efficacy of the exercise interventions with respect to the stated goals, per-protocol analysis was performed taking into account all subjects with a >90% attendance record for the exercise sessions. A sensitivity analysis (i.e., intention to treat analysis) was also performed using ‘baseline observation carried forward’ (BOCF) imputation for missing data.

Analysis of covariance (ANCOVA) was performed to examine the influence of the groups (fixed factor) on dependent outcomes, adjusting for baseline values (i.e., after intervention-cardiometabolic risk score minus baseline-cardiometabolic risk score). Bonferroni post hoc adjustment for multiple comparisons was used to examine differences between pairs of groups. Similar analyses were performed adjusting for age and sex as confounding variables.

Spearman correlation coefficients were also calculated to study the relationships between changes in the cardiometabolic risk score and QUICKI and HOMA values, and those in body composition, cardiorespiratory fitness, and dietary variables potentially modifiable by exercise.

Calculations were performed using the Statistical Package for the Social Sciences v.22.0 (IBM Corporation, Chicago, IL, USA). GraphPad Prism 5 software (GraphPad Software, San Diego, CA, USA) was used for plotting graphs. Significance was set at *p* ≤ 0.05.

## 3. Results

**Figure 1:**
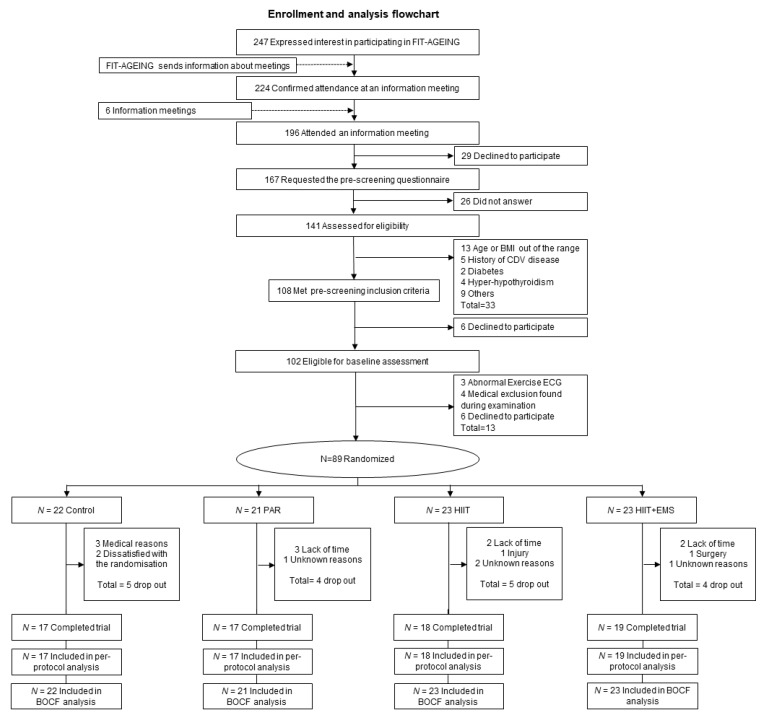
Enrolment and analysis flow-chart. Abbreviations: BMI—body mass index; CDV—cardiovascular disease; ECG—electrocardiogram; PAR—physical activity recommendations for adults’ group; HIIT—high intensity interval training group; HIIT+EMS—HIIT plus whole-body electromyostimulation group; QUICKI—quantitative insulin sensitivity check index; BOCF—baseline observation carried forward imputation.

Figure 1 shows the flowchart for enrolment and analysis. A total of 71 participants (*n* = 17 in the control group, *n* = 17 in the PAR group, *n* = 18 in the HIIT group and *n* = 19 in the HIIT+EMS group) completed the study. Table 1 shows the descriptive characteristics of the study subjects at baseline; no significant differences between groups were noted at this time.

**Figure 2:**
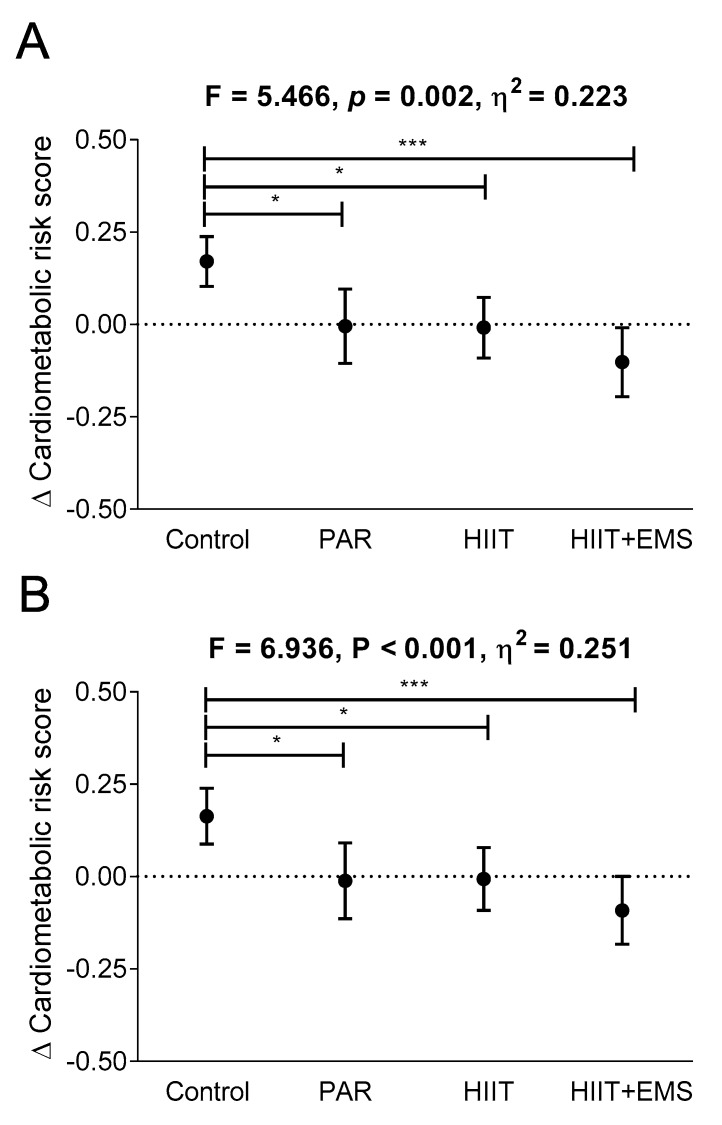
Changes in cardiometabolic risk after the intervention study in the four groups. Analysis of covariance adjusting for baseline values, with post hoc Bonferroni-corrected *t*-test (Panel **A**). Sensitivity analysis: baseline-observation carried forward imputation (Panel **B**). * *p <* 0.05, *** *p <* 0.001. The data are shown as means (standard deviation). Abbreviations: PAR—physical activity recommendations group; HIIT—high intensity interval training group; HIIT+EMS—HIIT plus whole-body electromyostimulation group.

Figure 2 shows changes in cardiometabolic risk score after the different exercise interventions. Compared to the control group, the cardiometabolic risk score decreased in the PAR, HIIT, and the HIIT+EMS groups (*p =* 0.026, *p* = 0.041, and *p <* 0.001, respectively; Figure 2A) with no significant differences between the three groups (all *p* > 0.5, Figure 2A). However, the HIIT+EMS group experienced the most significant reduction (−0.175 in the PAR group versus −0.179 in the HIIT group versus −0.272 in the HIIT-EMS group; *p <* 0.001; Figure 2B).

**Figure 3:**
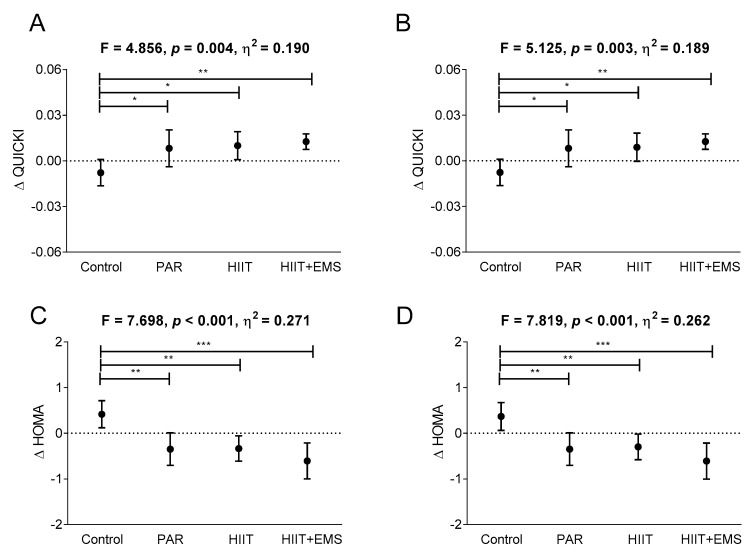
Changes in QUICKI (quantitative insulin sensitivity check index) and HOMA (homeostasis model assessment index) after the intervention study in the four groups. The *p* value is for ANCOVA adjusting for baseline values, with post hoc Bonferroni-corrected *t*-test (Panels **A** and **C**). Sensitivity analysis: baseline-observation carried forward imputation (Panels **B** and **D**). * *p <* 0.05, ** *p <* 0.01, *** *p <* 0.001. The data are shown as means (standard deviation). Abbreviations: PAR—physical activity recommendations group; HIIT—high intensity interval training group; HIIT+EMS—HIIT plus whole-body electromyostimulation group.

Figure 3 shows changes in QUICKI and HOMA indices after the intervention. Compared to the control group, the QUICKI index increased significantly in all exercise intervention groups (*p* = 0.026 for the PAR group, *p* = 0.016 for the HIIT group, and *p* = 0.010 for HIIT+EMS, respectively; Figure 3A), while their HOMA values fell significantly compared to the control group (*p* = 0.002 for PAR, *p* = 0.002 for HIIT, and *p* = 0.001 for HIIT+EMS, respectively; Figure 3C). No significant differences were seen among the exercise intervention groups (Figure 3A,C).

All the above findings persisted when sex and age were included as covariates (see Supplementary Table S3). Moreover, they remained consistent after performing BOCF sensitivity analysis (Figure 1B and Figure 2B,D and Supplementary Table S4). In addition, our findings did not change after performing the same analyses including the menopausal status of women as a confounding factor. Table 2 and Supplementary Table S5] show the changes recorded in anthropometric, blood pressure, glycaemic and lipid metabolism, and liver function variables.

A significant, negative relationship was detected between the change in lean mass and the change in cardiometabolic risk score (*p* = 0.045; Table 3), whereas no significant relationship was between the latter and a change in any other body composition, cardiorespiratory fitness or dietary variable (all *p* > 0.08; Table 3). Similarly, no significant correlations were observed between changes in body composition variables, cardiorespiratory fitness or dietary variables, and changes in the QUICKI or HOMA indices (all *p* > 0.05; Table 3).

## 4. Discussion

The main finding of this work is that, compared to a non-exercise control group, a 12-week supervised exercise training intervention improves cardiometabolic risk in sedentary middle-aged adults. It should be noted that, although the PAR and HIIT groups experienced reductions in cardiometabolic risk, the improvement seen for the HIIT+EMS group could be clinically greater. In addition, improvement in lean mass was significantly associated with a reduction in cardiometabolic risk, but no significant correlations were observed between the latter and changes in cardiorespiratory fitness or dietary variables. Taken together, these findings suggest that exercise training—especially a combination of HIIT and WB-EMS—improves cardiometabolic health in previously sedentary, middle-aged adults, independent of sex, age, or cardiorespiratory fitness.

It has been reported that concurrent training can lead to cardiometabolic benefits such as reductions in waist circumference, total cholesterol, LDL-C, triglycerides, plasma glucose, and blood pressure, and an increase in HDL-C [13,36,37,38,39,40]. In the present study, HDL-C increased, and both total cholesterol and blood pressure decreased in the PAR group, with the changes significantly larger than those recorded for the control group. These findings agree with those of other studies involving similar exercise training interventions [13,36,37,38]. It should be noted that no significant differences were seen between the PAR group and the control group with respect to the change in plasma glucose concentration. However, a significant difference was seen in the change in insulin sensitivity between these two groups (higher in the PAR group). Previous studies have suggested that exercise leads to improvements in plasma glucose when the baseline levels are higher than desirable [36], but in the present work, the mean baseline plasma glucose concentration of both groups was relatively normal.

It has been reported that HIIT helps reduce a number of cardiometabolic risk factors, including blood pressure [41], insulin sensitivity [42], and lipogenesis [42] in individuals with different biological characteristics. A recent systematic review and meta-analysis indicated that HIIT may be a time-efficient training method in terms of improving cardiometabolic health, providing similar improvements to those achieved with continuous endurance training at moderate intensity [43]. These findings agree with those of the present study, with improvements of the same magnitude obtained in the PAR and HIIT groups.

A study that examined the effects of combining WB-EMS and whey protein supplementation on cardiometabolic risk in men aged over 70 years with sarcopenic obesity, reported a significant improvement in cardiometabolic risk after 16 weeks [18]. However, this study did not answer what the effects of an WB-EMS program without whey protein supplementation might be, what the effects might be of a WB-EMS program on the cardiometabolic profile of sedentary men or women under 70 years of age, or whether an HIIT program plus WB-EMS might produce additional improvements in cardiometabolic risk compared with those obtained by an HIIT program without WB-EMS or with any other type of exercise training. The present work shows that an HIIT program plus WB-EMS can significantly improve the cardiometabolic profile, at least in previously sedentary, middle-aged adults compared to controls. Interestingly, although no significant differences in cardiometabolic profile were observed between the HIIT+EMS group and the HIIT or PAR groups after the corresponding interventions, a clinically relevant reduction in cardiometabolic risk was noted in the change in the HIIT+EMS group compared to the other exercise training groups, independent of sex age, or cardiorespiratory fitness. These findings suggest that an HIIT plus WB-EMS might be the most effective training methodology for improving the cardiometabolic profile-perhaps even more so than concurrent training based on the international physical activity guidelines (which involves a higher exercise volume and frequency) [11,12].

The additional cardiometabolic improvements obtained by the HIIT+EMS group might be the consequence of the larger number of muscular contractions leading to a greater increase in lean mass [20]. Previous studies have proposed the physiological mechanisms via which an enhanced muscular mass might reduce the incidence of chronic cardiometabolic disease [13,44,45]. Skeletal muscle can be regarded as an endocrine organ since, in response to contraction, it produces myokines-molecules that play a crucial role in the modulation of obesity, metabolic syndrome, and type II diabetes mellitus [46]. It is therefore plausible that exercise-induced changes in lean mass can reduce cardiometabolic risk. The present results partially support this notion; a significant negative relationship was seen between the change in lean mass and the changes in cardiometabolic risk, but no other significant relationships were observed between changes in fat mass or visceral adipose tissue with changes in cardiometabolic risk.

It has been well documented that high cardiorespiratory fitness is associated with a reduced risk of chronic cardiometabolic disease [8]. However, in addition to enhancing the former, a well-designed exercise training program should have favorable effects on glucose and lipid metabolism, and on blood pressure [10]. Certainly, some controversy surrounds the impact of changes in cardiorespiratory fitness on cardiometabolic risk, with some studies reporting an improvement to be a significant predictor of an improved glycaemic and lipid profiles [47,48,49], while others report no such association at all [50,51]. However, the majority of studies has been conducted in patients with cardiometabolic diseases, and has commonly involved individuals with type II diabetes mellitus. It has remained unclear whether exercise training-induced changes in cardiorespiratory fitness are related to changes in cardiometabolic risk, since sedentary, middle-aged people naturally have age-related increased risk of developing cardiometabolic problems [52]. The current study identified significant improvements in cardiometabolic risk for all the treatment groups, independent of changes in cardiorespiratory fitness, and even though a significant increase in cardiorespiratory fitness was seen [19]. The present lack of any association between changes in cardiorespiratory fitness and improvements in cardiometabolic profile might be explained in that exercise promotes a number of adaptive mechanisms [53]. While the enhancement of cardiorespiratory fitness in response to exercise is predominantly related to central cardiovascular adaptations, heart remodeling and an increase in stroke volume, training-associated changes in cardiometabolic profile are more related to improvements in insulin sensitivity caused by specific adaptations in adipose and skeletal muscle [10,50], an argument that the present findings support.

The present work suffers from a number of limitations. The standard deviation for some variables was higher than expected; therefore, the work may be underpowered for detecting statistical differences between the exercise training groups with respect to some dependent outcomes. Furthermore, insulin sensitivity/resistance was not determined by the gold standard method (i.e., the hyperinsulinemic euglycemic glucose clamp technique). However, both the QUICKI [32] and HOMA [33] methods have been validated for assessing insulin sensitivity and insulin resistance, respectively.

## 5. Conclusions

In conclusion, the finding of this work suggests that a supervised exercise training intervention (independently of its modality) improves cardiometabolic risk compared with a non-exercise control group in sedentary middle-aged adults independent of sex, age and cardiorespiratory fitness. Of interest is that, although the PAR and HIIT groups experienced reductions in cardiometabolic risk, the improvement seen for the HIIT+EMS group could be clinically greater. These results have important clinical implications: while the training intervention based on international physical activity guidelines (PAR group) improved cardiometabolic risk compared to a non-exercise control treatment, the HIIT+EMS program seems to induce slightly better results with less than half the training volume. Since the majority of individuals in developed countries do not meet current international physical activity recommendations, largely through a lack of time, this type of training might be particularly valuable. Further studies should be conducted to confirm these findings and to determine whether the same holds true for other populations.

## Figures and Tables

**Table 1:** Baseline descriptive characteristics of the study subjects included in the per-protocol analysis.

	All (*n* = 71)	Control (*n* = 17)	PAR (*n* = 17)	HIIT (*n* = 18)	HIIT + EMS (*n* = 19)	*p* Value
Age (years)	53.42 (4.91)	52.09 (4.05)	54.92 (4.54)	53.14 (5.59)	53.53 (5.25)	0.414
Sex (%)						
Men	34 (47.9)	7 (41.2)	8 (47.1)	9 (50.0)	10 (52.6)	
Women	37 (52.1)	10 (58.8)	9 (52.9)	9 (50.0)	9 (47.4)	
Anthropometry and Body Composition						
Body mass index (kg/m^2^)	26.82 (3.79)	26.67 (3.71)	25.41 (2.86)	26.43 (3.15)	28.60 (4.64)	0.077
Waist circumference (cm)	95.29 (11.89)	93.35 (10.37)	90.43 (11.01)	97.53 (10.88)	99.26 (13.69)	0.107
Fat mass (kg)	30.15 (8.39)	28.64 (6.84)	26.83 (6.31)	31.42 (8.30)	33.27 (10.36)	0.097
Fat mass (%)	39.75 (8.78)	39.39 (9.30)	37.38 (8.78)	40.74 (8.56)	41.26 (8.75)	0.570
Visceral adipose tissue (g)	788.9 (391.8)	710.6 (272.4)	661.3 (262.6)	813.6 (452.2)	949.8 (477.1)	0.122
Lean mass (kg)	43.92 (11.59)	42.92 (12.06)	43.60 (10.77)	44.43 (13.52)	44.60 (10.76)	0.972
Blood Pressure						
Systolic blood pressure (mm Hg)	127.09 (15.78)	127.00 (18.45)	128.88 (13.36)	126.72 (16.68)	125.88 (15.49)	0.959
Diastolic blood pressure (mm Hg)	81.12 (11.72)	82.38 (14.54)	81.75 (10.96)	80.50 (11.39)	80.0 (10.70)	0.936
Mean blood pressure (mm Hg)	104.10 (13.15)	104.69 (16.00)	105.31 (11.36)	103.61 (13.78)	102.94 (12.13)	0.957
Glucose Metabolism						
Plasma glucose (mg/dL)	93.56 (11.36)	93.47 (10.82)	93.35 (11.63)	90.06 (5.56)	96.95 (14.80)	0.352
Plasma insulin (UI/mL)	8.08 (5.68)	7.26 (5.05)	7.52 (3.97)	7.09 (4.51)	10.22 (7.88)	0.296
Insulin glucose ratio	12.58 (7.56)	11.22 (6.73)	12.02 (6.23)	11.82 (7.05)	14.98 (9.57)	0.442
QUICKI	0.362 (0.036)	0.366 (0.035)	0.361 (0.032)	0.370 (0.037)	0.350 (0.040)	0.402
HOMA	1.93 (1.67)	1.73 (1.37)	1.75 (0.99)	1.59 (1.05)	2.59 (2.55)	0.255
Lipid Metabolism						
Total cholesterol (mg/dL)	206.14 (32.17)	201.47 (33.98)	204.11 (17.73)	214.06 (43.34)	206.05 (28.87)	0.696
HDL-C (mg/dL)	58.71 (12.28)	61.06 (11.99)	55.18 (12.03)	57.82 (10.79)	60.58 (14.03)	0.473
LDL-C (mg/dL)	126.23 (27.07)	123.82 (28.00)	121.53 (19.74)	131.24 (35.93)	128.11 (23.77)	0.733
Triglycerides (mg/dL)	134.24 (68.16)	145.18 (81.62)	130.88 (70.00)	134.06 (61.48)	127.63 (63.27)	0.888
LDL-C/HDL-C	2.31 (0.90)	2.20 (1.01)	2.33 (0.70)	2.45 (1.12)	2.27 (0.79)	0.870
Triglycerides/HDL-C	2.57 (1.92)	2.68 (2.08)	2.67 (2.02)	2.58 (1.77)	2.37 (1.93)	0.961
Cardiometabolic Risk Score	−0.0002 (0.3414)	−0.0448 (0.3249)	−0.0254 (0.2822)	0.0039 (0.4164)	0.0615 (0.3460)	0.828
Liver Function						
ALT (IU/L)	23.14 (12.53)	24.41 (14.51)	22.18 (10.06)	20.71 (9.74)	25.05 (15.13)	0.724
γ-GT (IU/L)	33.99 (23.26)	36.76 (27.56)	30.47 (18.12)	28.29 (17.01)	39.74 (27.64)	0.429
Fatty liver index	50.12 (26.55)	49.04 (29.04)	39.74 (23.43)	50.46 (24.87)	60.06 (26.59)	0.151
Dietary Intake						
Energy (kcal/day)	2141 (699)	2079 (495)	2288 (1152)	2149 (514)	2054 (455)	0.767
Fat (g/day)	37.55 (6.90)	37.09 (9.20)	37.31 (8.03)	36.32 (5.93)	39.32 (4.08)	0.601
Carbohydrate (g/day)	47.14 (8.19)	49.82 (10.41)	47.85 (8.45)	47.17 (6.00)	44.21 (7.30)	0.236
Protein (g/day)	18.64 (4.91)	16.94 (4.35)	19.23 (6.84)	19.36 (4.90)	18.84 (2.97)	0.467
Ethanol (g/day)	10.57 (11.69)	9.43 (10.12)	9.70 (10.73)	10.64 (9.25)	12.23 (15.84)	0.894
Cardiorespiratory Fitness						
VO_2_max (mL/min)	2339.2 (657.2)	2163.4 (626.0)	2320.4 (649.7)	2461.8 (709.1)	2397.1 (658.3)	0.580
VO_2_max_weight_ (mL/kg/min)	30.49 (5.57)	28.99 (4.96)	31.64 (6.12)	31.59 (6.22)	29.74 (4.90)	0.399

Data are shown as means (standard deviation). Abbreviations: PAR—physical activity recommendations for adults group; HIIT—high intensity interval training group; HIIT+EMS—HIIT plus whole-body electromyostimulation group; QUICKI—quantitative insulin sensitivity check index; HOMA—homeostasis model assessment index; HDL-C—high-density lipoprotein cholesterol; LDL-C—low-density lipoprotein cholesterol; ALT—Alanine transaminase; γ-GT-γ—glutamyl transferase, VO_2_max—maximum oxygen uptake. *p* Value, one-way ANOVA (to detect between-group differences at baseline).

**Table 2:** Changes in anthropometric variables, blood pressure, glycaemic and lipid metabolism, and liver function after a 12-week intervention program.

Change from Baseline at Week 12	Intervention												F	*p* Value	η^2^
	Control (*n* = 17)			PAR (*n* = 17)			HIIT (*n* = 18)			HIIT+EMS (*n* = 19)					
	Pre	Post	Δ	Pre	Post	Δ	Pre	Post	Δ	Pre	Post	Δ			
Anthropometry															
Body mass index (kg/m^2^)	26.39 (3.80)	26.21 (3.65)	−0.18 (0.34)	25.41 (2.86)	24.90 (2.43)	−0.51 (0.66)	26.43 (3.15)	26.37 (2.96)	−0.06 (0.53)	28.60 (4.64)	28.40 (4.64)	−0.20 (0.42)	**3.993**	**0.011**	**0.160**
Waist circumference (cm)	92.46 (10.81)	92.30 (11.63)	−0.16 (2.12)	90.43 (11.01)	88.54 (10.23)	−1.90 (3.45)	97.53 (10.88)	93.00 (11.11)	−4.53 (2.54)	99.26 (13.69)	95.26 (13.79)	−4.00 (2.38)	**7.749**	**0.011**	**0.270**
Blood pressure															
Systolic blood pressure (mm Hg)	129.85 (15.17)	130.23 (15.50)	0.38 (2.47)	128.88 (13.36)	125.38 (11.78)	−3.5 (2.19)	126.72 (16.68)	124.67 (15.4)	−2.06 (2.1)	125.88 (15.49)	119.41 (13.24)	−6.47 (3.34)	**8.651**	**<0.001**	**0.593**
Diastolic blood pressure (mm Hg)	84.92 (9.45)	86.00 (10.44)	1.08 (2.63)	81.75 (10.96)	80.19 (9.81)	−1.56 (1.90)	80.50 (11.39)	79.33 (10.63)	−1.17 (1.92)	80.00 (10.70)	75.65 (8.46)	−4.35 (3.20)	**7.840**	**0.001**	**0.476**
Mean blood pressure (mm Hg)	107.39 (11.63)	108.12 (12.28)	0.73 (2.41)	105.31 (11.36)	102.78 (9.96)	−2.53 (1.82)	103.61 (13.78)	102.00 (12.73)	−1.61 (1.81)	102.94 (12.13)	97.53 (9.77)	−5.41 (3.14)	**27.422**	**0.001**	**0.582**
Glucose metabolism															
Plasma glucose (mg/dL)	94.87 (10.74)	93.73 (7.78)	−1.13 (7.75)	93.35 (11.63)	91.29 (9.42)	−2.06 (8.12)	89.75 (5.59)	90.31 (8.55)	0.56 (5.89)	96.95 (14.80)	92.89 (11.88)	−4.05 (6.28)	0.568	0.638	0.027
Plasma insulin (UI/mL)	7.03 (5.28)	8.96 (6.90)	1.93 (2.63)	7.52 (3.97)	6.15 (2.65)	−1.37 (3.01)	7.39 (4.48)	5.84 (3.03)	−1.55 (2.66)	10.22 (7.88)	8.34 (6.55)	−1.88 (2.05)	**7.357**	**<0.001**	**0.263**
Insulin glucose ratio	10.48 (6.70)	14.17 (9.61)	3.69 (4.86)	12.02 (6.23)	10.04 (3.70)	−1.99 (5.14)	12.26 (7.03)	9.66 (4.69)	−2.59 (4.47)	14.98 (9.57)	13.12 (8.85)	−1.87 (3.11)	**6.474**	**0.001**	**0.239**
QUICKI	0.37 (0.04)	0.36 (0.04)	−0.01 (0.02)	0.36 (0.03)	0.37 (0.03)	0.01 (0.02)	0.37 (0.03)	0.38 (0.03)	0.01 (0.02)	0.35 (0.04)	0.36 (0.04)	0.01 (0.01)	**4.856**	**0.004**	**0.190**
HOMA	1.71 (1.45)	2.13 (1.74)	0.42 (0.62)	1.75 (0.99)	1.40 (0.69)	−0.35 (0.69)	1.65 (1.05)	1.32 (0.72)	−0.33 (0.59)	2.59 (2.55)	1.98 (1.83)	−0.61 (0.82)	**7.696**	**<0.001**	**0.271**
Lipid metabolism															
Total cholesterol (mg/dL)	200.53 (36.13)	206.67 (30.83)	6.13 (38.33)	204.12 (17.73)	203.12 (21.30)	−1.00 (19.49)	217.56 (42.2)	214.44 (38.76)	−3.13 (36.54)	206.05 (28.87)	190.74 (27.60)	−15.32 (12.17)	2.230	0.093	0.097
HDL-C (mg/dL)	61.20 (12.12)	60.53 (10.76)	−0.67 (11.88)	55.18 (12.03)	59.88 (15.59)	4.71 (10.95)	57.69 (11.13)	62.81 (17.03)	5.13 (12.93)	60.58 (14.03)	62.79 (18.03)	2.21 (12.82)	0.536	0.660	0.032
LDL-C (mg/dL)	124.53 (29.54)	128.13 (31.65)	3.60 (35.82)	121.53 (19.74)	125.76 (22.76)	4.24 (21.14)	133.38 (35.97)	137.94 (38.26)	4.56 (28.84)	128.11 (23.77)	110.05 (27.72)	−18.05 (18.88)	**3.562**	**0.019**	**0.147**
Triglycerides (mg/dL)	131.20 (72.33)	134.47 (108.83)	3.27 (57.84)	130.88 (70.00)	104.18 (55.83)	−26.71 (60.07)	135.94 (62.99)	120.50 (71.99)	−15.44 (60.42)	127.63 (63.27)	97.21 (49.54)	−30.42 (41.10)	**3.869**	**0.013**	**0.158**
LDL-C/HDL-C	2.21 (1.05)	2.19 (0.71)	−0.01 (1.13)	2.33 (0.70)	2.19 (0.52)	−0.14 (0.57)	2.50 (1.14)	2.40 (1.06)	−0.10 (0.75)	2.27 (0.79)	1.95 (0.87)	−0.33 (0.55)	0.920	0.436	0.043
Triglycerides/HDL-C	2.44 (1.97)	2.41 (2.30)	−0.03 (1.38)	2.67 (2.02)	1.91 (1.38)	−0.76 (1.55)	2.63 (1.82)	2.24 (1.78)	−0.39 (1.38)	2.37 (1.93)	1.84 (1.51)	−0.53 (1.09)	0.929	0.432	0.043
Cardiometabolic risk score	−0.04 (0.32)	0.13 (0.29)	0.17 (0.14)	−0.03 (0.28)	−0.03 (0.24)	0.01 (0.20)	0.01 (0.43)	0.00 (0.39)	−0.01 (0.18)	0.06 (0.35)	−0.04 (0.32)	−0.10 (0.20)	**5.466**	**0.002**	**0.226**
Liver function															
ALT (IU/L)	24.60 (15.38)	25.07 (14.84)	0.47 (7.85)	22.18 (10.06)	21.65 (8.99)	−0.53 (6.76)	20.06 (9.68)	23.31 (13.02)	3.25 (7.02)	25.05 (15.13)	25.84 (12.69)	0.79 (8.72)	0.594	0.621	0.028
γ-GT (IU/L)	39.07 (28.64)	36.87 (30.05)	−2.20 (5.87)	30.47 (18.12)	27.65 (13.84)	−2.82 (7.88)	28.38 (17.56)	28.63 (16.18)	0.25 (5.16)	39.74 (27.64)	39.21 (26.06)	−0.53 (10.17)	0.680	0.568	0.032
Fatty liver index	45.70 (29.62)	42.48 (30.16)	−3.22 (7.13)	39.74 (23.44)	30.89 (15.94)	−8.85 (12.99)	49.32 (25.22)	41.60 (23.74)	−7.72 (9.04)	60.06 (26.6)	49.83 (29.91)	−10.24 (10.51)	1.167	0.330	0.054

Abbreviations: PAR—physical activity recommendations for adults group; HIIT—high intensity interval training group; HIIT+EMS—HIIT plus whole-body electromyostimulation group; QUICKI—quantitative insulin sensitivity check index; HOMA—homeostasis model assessment index; HDL-C—high-density lipoprotein cholesterol; LDL-C—low-density lipoprotein cholesterol; ALT—Alanine transaminase; γ-GT-γ—glutamyl transferase, VO_2_max—maximum oxygen uptake. *p* Value, one-way ANOVA (to detect between-group differences at baseline). *p* Value for analysis of covariance adjusting for baseline, with post hoc Bonferroni-corrected *t*-test (similar letters indicate significant differences). The bold *p* values mean significant differences.

**Table 3:** Spearman correlation coefficients (R_s_) between changes in cardiometabolic risk, fatty liver index, Quantitative insulin sensitivity check (QUICKI) index, and homeostasis model assessment (HOMA) index, and body composition, cardiorespiratory fitness (maximum oxygen uptake [VO2max]), and dietary variables (excluding control group).

	Δ Cardiometabolic Risk Score		Δ QUICKI Index		Δ HOMA Index	
	R_s_	*p* Value	R_s_	*p* Value	R_s_	*p* Value
Δ Fat mass (%)	0.258	0.083	−0.043	0.769	0.155	0.287
Δ Visceral adipose tissue (g)	0.227	0.130	−0.010	0.944	0.200	0.167
Δ Lean mass (kg)	**−0.291**	**0.045**	0.071	0.629	−0.173	0.235
Δ VO_2_max (mL/kg/min)	−0.108	0.461	−0.125	0.376	0.085	0.548
Δ Energy intake (kcal/day)	−0.018	0.902	−0.072	0.615	−0.027	0.852
Δ Fat (g/day)	−0.032	0.829	0.168	0.244	−0.101	0.485
Δ Carbohydrate (g/day)	0.054	0.719	0.082	0.572	−0.040	0.781
Δ Protein (g/day)	−0.206	0.164	−0.167	0.245	0.076	0.599
Δ Ethanol (g/day)	0.087	0.561	0.029	0.843	0.015	0.919

The bold values mean significant differences.

## Supplementary Materials

The following are available online at www.mdpi.com/xxx/s1,Table S1: CONSORT 2010 checklist of information to include when reporting a randomised trial, Table S2: CERT checklist, Table S3: Changes in anthropometric variables, blood pressure, glucose and lipid metabolism, cardiometabolic risk score, and liver function adjusted for baseline values and sex (Model 1), and adjusted for baseline values and age (Model 2), Table S4: Sensitivity (intention to treat) analyses: baseline-observation carried forward imputation assessing the effects of a 12-week intervention program on anthropometric variables, blood pressure, glucose and lipid metabolism, cardiometabolic risk, and liver function, adjusted by baseline values and sex (Model 1), and adjusted by baseline values and age (Model 2), Table S5: Sensitivity analysis: baseline-observation carried forward imputation assessing the effects of a 12-week intervention program on anthropometric variables, blood pressure, glucose and lipid metabolism, and liver function.
