# Supplementary material for: Exercise Training as a Treatment for Cardiometabolic Risk in Sedentary Adults: Are Physical Activity Guidelines the Best Way to Improve Cardiometabolic Health? The FIT-AGEING Randomized Controlled Trial
Source: J Clin Med. 2019 Dec 1;8(12):2097. doi: 10.3390/jcm8122097 (PMC6947226; doi:10.3390/jcm8122097)
Supplement: Supplementary file 1 [file jcm-08-02097-s001.pdf]

Supplementary Table S1. CONSORT 2010 checklist of information to include when reporting a randomised trial

CONSORT 2010 checklist of information to include when reporting a randomised trial*

Section/Topic	Item No	Checklist item	Reported on page No
**Title and abstract**			
	1a	Identification as a randomised trial in the title	1
	1b	Structured summary of trial design, methods, results, and conclusions (for specific guidance see CONSORT for abstracts)	2
**Introduction**			
Background and objectives	2a	Scientific background and explanation of rationale	4-5
	2b	Specific objectives or hypotheses	5
**Methods**			
Trial design	3a	Description of trial design (such as parallel, factorial) including allocation ratio	6
	3b	Important changes to methods after trial commencement (such as eligibility criteria), with reasons	6-8
Participants	4a	Eligibility criteria for participants	6
	4b	Settings and locations where the data were collected	6-8
Interventions	5	The interventions for each group with sufficient details to allow replication, including how and when they were actually administered	6-8
Outcomes	6a	Completely defined pre-specified primary and secondary outcome measures, including how and when they were assessed	8-10
	6b	Any changes to trial outcomes after the trial commenced, with reasons	Not applicable
Sample size	7a	How sample size was determined	10-11
	7b	When applicable, explanation of any interim analyses and stopping guidelines	10-12
Randomisation:			

Sequence generation	8a	Method used to generate the random allocation sequence	6
	8b	Type of randomisation; details of any restriction (such as blocking and block size)	6
Allocation concealment mechanism	9	Mechanism used to implement the random allocation sequence (such as sequentially numbered containers), describing any steps taken to conceal the sequence until interventions were assigned	6
Implementation	10	Who generated the random allocation sequence, who enrolled participants, and who assigned participants to interventions	6-8
Blinding	11a	If done, who was blinded after assignment to interventions (for example, participants, care providers, those assessing outcomes) and how	6-8
	11b	If relevant, description of the similarity of interventions	6-8
Statistical methods	12a	Statistical methods used to compare groups for primary and secondary outcomes	10-12
	12b	Methods for additional analyses, such as subgroup analyses and adjusted analyses	10-12
**Results**			
Participant flow (a diagram is strongly recommended)	13a	For each group, the numbers of participants who were randomly assigned, received intended treatment, and were analysed for the primary outcome	13
	13b	For each group, losses and exclusions after randomisation, together with reasons	13
Recruitment	14a	Dates defining the periods of recruitment and follow-up	7
	14b	Why the trial ended or was stopped	Not applicable
Baseline data	15	A table showing baseline demographic and clinical characteristics for each group	13
Numbers analysed	16	For each group, number of participants (denominator) included in each analysis and whether the analysis was by original assigned groups	13
Outcomes and estimation	17a	For each primary and secondary outcome, results for each group, and the estimated effect size and its precision (such as 95% confidence interval)	13-15
	17b	For binary outcomes, presentation of both absolute and relative effect sizes is recommended	13-15
Ancillary analyses	18	Results of any other analyses performed, including subgroup analyses and adjusted analyses, distinguishing pre-specified from exploratory	13-15

Harms	19	All-important harms or unintended effects in each group (for specific guidance see CONSORT for harms)	13-15
**Discussion**			
Limitations	20	Trial limitations, addressing sources of potential bias, imprecision, and, if relevant, multiplicity of analyses	19
Generalisability	21	Generalisability (external validity, applicability) of the trial findings	16-20
Interpretation	22	Interpretation consistent with results, balancing benefits and harms, and considering other relevant evidence	16-20
**Other information**			
Registration	23	Registration number and name of trial registry	6
Protocol	24	Where the full trial protocol can be accessed, if available	6
Funding	25	Sources of funding and other support (such as supply of drugs), role of funders	21

*We strongly recommend reading this statement in conjunction with the CONSORT 2010 Explanation and Elaboration for important clarifications on all the items. If relevant, we also recommend reading CONSORT extensions for cluster randomised trials, non-inferiority and equivalence trials, non-pharmacological treatments, herbal interventions, and pragmatic trials. Additional extensions are forthcoming: for those and for up to date references relevant to this checklist, see www.consort-statement.org.

Supplementary Table S2. CERT checklist.

	Item	Checklist Item	Identification
**WHAT: materials**	1	Detailed description of the type of exercise equipment	6-8
**WHO: provider**	2	Detailed description of the qualifications, expertise and/or training	8
**HOW: delivery**	3	Describe whether exercises are performed individually or in a group	7-8
	4	Describe whether exercises are supervised or unsupervised; how they are delivered	8
	5	Detailed description of how adherence to exercise is measured and reported	6-8
	6	Detailed description of motivation strategies	6-8
	7a	Detailed description of the decision rule (s) for determining exercise progression	6-8
	7b	Detailed description of how the exercise program was progressed	6-8
	8	Detailed description of each exercise to enable replication	7-8
	9	Detailed description of any home programme component	Not applicable
	10	Describe whether there are any non-exercise components	6
	11	Describe the type and number of adverse events that occur during exercise	Not applicable
**WHERE: location**	12	Describe the setting in which the exercises are performed	6-8
**WHEN, HOW MUCH: dosage**	13	Detailed description of the exercise intervention	7-8
**TAILORING: what, how**	14a	Describe whether the exercises are generic (one size fits all) or tailored	7-8
	14b	Detailed description of how exercises are tailored to the individual	7-8
	15	Describe the decision rule for determining the starting level	7-8
**HOW WELL: planned, actual**	16a	Describe how adherence or fidelity is assessed/measured	6-8
	16b	Describe the extent to which the intervention was delivered as planned	6-8

Supplementary Table S3. Changes in anthropometric variables, blood pressure, glucose and lipid metabolism, cardiometabolic risk score, and liver function adjusted for baseline values and sex (Model 1), and adjusted for baseline values and age (Model 2).

	**Analysis of covariance P value**	
	**Model 1**	**Model 2**
Anthropometry		
Body mass index (kg/m^2)	**0.013**	**0.005**
Waist circumference (cm)	**<0.001**	**<0.001**
Blood pressure		
Systolic blood pressure (mm Hg)	**<0.001**	**<0.001**
Diastolic blood pressure (mm Hg)	**<0.001**	**<0.001**
Mean blood pressure (mm Hg)	**<0.001**	**<0.001**
Glucose metabolism		
Plasma glucose (mg/dL)	0.691	0.671
Plasma insulin (UI/mL)	**<0.001**	**<0.001**
Insulin glucose ratio	**0.001**	**0.001**
QUICKI	**0.005**	**0.008**
HOMA	**<0.001**	**<0.001**
Lipid metabolism		
Total cholesterol (mg/dL)	0.116	0.079
HDL-C (mg/dL)	0.623	0.708
LDL-C (mg/dL)	**0.024**	**0.020**
Triglycerides (mg/dL)	0.097	0.264
LDL-C/HDL-C	0.368	0.421
Triglycerides/HDL-C	0.310	0.425
Cardiometabolic risk score	**0.002**	**0.003**
Liver function		
ALT (IU/L)	0.619	0.633
γ-GT (IU/L)	0.575	0.578
Fatty liver index	0.282	0.364

Abbreviations: QUICKI - quantitative insulin sensitivity check index; HOMA - homeostasis model assessment index; HDL-C - high-density lipoprotein cholesterol; LDL-C -low-density lipoprotein cholesterol; ALT - alanine transaminase, γ-GT - γ-glutamyl transferase.

Supplementary Table S4. Sensitivity (intention to treat) analyses: baseline-observation carried forward imputation assessing the effects of a 12-week intervention program on anthropometric variables, blood pressure, glucose and lipid metabolism, cardiometabolic risk, and liver function, adjusted by baseline values and sex (Model 1), and adjusted by baseline values and age (Model 2).

	**ANCOVA P value**	
	**Model 1**	**Model 2**
Anthropometry		
Body mass index (kg/m^2)	**0.010**	**0.004**
Waist circumference (cm)	**<0.001**	**<0.001**
Blood pressure		
Systolic blood pressure (mm Hg)	**<0.001**	**<0.001**
Diastolic blood pressure (mm Hg)	**<0.001**	**<0.001**
Mean blood pressure (mm Hg)	**<0.001**	**<0.001**
Glucose metabolism		
Plasma glucose (mg/dL)	0.654	0.683
Plasma insulin (UI/mL)	**<0.001**	**0.001**
Insulin glucose ratio	**<0.001**	**0.001**
QUICKI	**0.003**	**0.005**
HOMA	**<0.001**	**<0.001**
Lipid metabolism		
Total cholesterol (mg/dL)	**0.116**	**0.079**
HDL-C (mg/dL)	0.623	0.708
LDL-C (mg/dL)	**0.024**	**0.020**
Triglycerides (mg/dL)	0.190	0.264
LDL-C/HDL-C	0.396	0.421
Triglycerides/HDL-C	0.310	0.425
Cardiometabolic risk score	**0.002**	**0.003**
Liver function		
ALT (IU/L)	0.613	0.645
γ-GT (IU/L)	0.532	0.538
Fatty liver index	0.282	0.364

Abbreviations: QUICKI - quantitative insulin sensitivity check index; HOMA - homeostasis model assessment index; HDL-C - high-density lipoprotein cholesterol; LDL-C -low-density lipoprotein cholesterol; ALT - alanine transaminase, γ-GT - γ-glutamyl transferase.

Supplementary Table S5. Sensitivity analysis: baseline-observation carried forward imputation assessing the effects of a 12-week intervention program on anthropometric variables, blood pressure, glucose and lipid metabolism, and liver function.

Change from Baseline at week 12	Intervention				F	P value	η^2
	Control (n=17) Mean change (SD)	PAR (n=17) Mean change (SD)	HIIT (n=18) Mean change (SD)	HIIT+EMS (n=19) Mean change (SD)			
Anthropometry							
Body mass index (kg/m^2)	-0.15 (0.32)	-0.51 (0.65)[ab]	-0.06 (0.53)[a]	-0.20 (0.51)[b]	**4.184**	**0.009**	**0.160**
Waist circumference (cm)	-0.13 (1.91)[ab]	-1.90 (3.45)[c]	-4.53 (2.54)[ac]	-4.00 (2.38)[b]	**9.318**	**<0.001**	**0.298**
Blood pressure							
Systolic blood pressure (mm Hg)	0.38 (2.46)[abc]	-3.50 (2.19)[ad]	-2.06 (2.10)[be]	-6.47 (3.34)[cde]	**28.651**	**<0.001**	**0.593**
Diastolic blood pressure (mm Hg)	1.08 (2.62)[abc]	-1.56 (1.90)[ad]	-1.17 (1.92)[be]	-4.35 (3.20)[cde]	**17.840**	**<0.001**	**0.476**
Mean blood pressure (mm Hg)	0.73 (2.41)[abc]	-2.53 (1.82)[ad]	-1.61 (1.81)[be]	-5.41 (3.14)[cde]	**27.422**	**<0.001**	**0.582**
Glucose metabolism							
Plasma glucose (mg/dL)	-1.00 (7.26)[abc]	-2.06 (8.12)[a]	0.50 (5.53)[b]	-4.05 (6.28)[c]	0.558	0.644	0.025
Plasma insulin (UI/mL)	1.70 (2.55)[abc]	-1.37 (3.01)[a]	-1.37 (2.54)[b]	-1.88 (2.05)[c]	**7.434**	**<0.001**	**0.253**
Insulin glucose ratio	3.25 (4.70)[abc]	-1.98 (5.14)[a]	-2.30 (4.27)[b]	-1.87 (3.11)[c]	**6.642**	**0.001**	**0.232**
Lipid metabolism							
Total cholesterol (mg/dL)	6.13 (38.33)	-1.00 (19.49)	-3.13 (36.54)	-15.32 (12.17)[b]	2.230	0.093	0.097
HDL-C (mg/dL)	-0.67 (11.88)	4.71 (10.95)	5.12 (12.92)	2.21 (12.82)	0.536	0.660	0.025
LDL-C (mg/dL)	3.60 (35.82)[a]	4.24 (21.14)	4.56 (28.84)[b]	-18.05 (18.88)[ab]	**3.562**	**0.019**	**0.147**
Triglycerides (mg/dL)	3.27 (57.83)	-26.71 (60.07)	-15.44 (60.41)	-30.42 (41.10)	1.387	0.255	0.063
LDL-C/HDL-C	0.01 (1.13)	-0.14 (0.57)	-0.10 (0.75)	-0.33 (0.55)	0.920	0.436	0.043
Triglycerides/HDL-C	-0.03 (1.38)	-0.76 (1.55)	-0.39 (1.37)	-0.53 (1.08)	0.929	0.432	0.043
Liver function							
ALT (IU/L)	0.41 (7.35)	-0.53 (6.77)	2.88 (6.68)	0.79 (8.72)	0.757	0.633	0.025
γ-GT (IU/L)	-1.94 (5.54)	-2.82 (7.88)	0.22 (4.84)	-0.53 (10.17)	0.754	0.524	0.033
Fatty liver index	-3.07 (7.01)	-8.71 (12.25)	-7.64 (9.00)	-10.10 (10.27)	1.214	0.338	0.051

P value for ANCOVA adjusting for baseline, with post hoc Bonferroni-corrected t test (same letters indicate significant differences). Abbreviations: PAR - physical activity recommendations group; HIIT - high intensity interval training group; HIIT+EMS - whole-body electromyostimulation group; ALT - Alanine transaminase; γ-GT - γ-glutamyl transferase. P value for one way ANOVA to detect differences between groups at baseline.
